# The proportion of HIV disclosure to sexual partners among people diagnosed with HIV in China: A systematic review and meta-analysis

**DOI:** 10.3389/fpubh.2022.1004869

**Published:** 2022-10-17

**Authors:** Wenwen Peng, Xiaohan Song, Ci Zhang, Yuqing Chen, Qidi Zhou, Maritta Anneli Välimäki, Xianhong Li

**Affiliations:** ^1^Xiangya School of Nursing, Central South University, Changsha, Hunan Province, China; ^2^Xiangya Center for Evidence-Based Nursing Practice & Healthcare Innovation (A JBI Affiliated Group), Changsha, Hunan Province, China; ^3^Department of Nursing Science, University of Turku, Turku, Finland

**Keywords:** HIV disclosure, sexual partners, people diagnosed with HIV, China, systematic review and meta-analysis

## Abstract

**Background:**

Sexual behavior is one of the main routes of HIV/AIDS spread. HIV disclosure to sexual partners has been confirmed to be an important strategy for HIV/AIDS prevention and control. We conducted a systematic review and meta-analysis to pool proportions and characteristics of HIV disclosure to sexual partners among people diagnosed with HIV in China.

**Methods:**

We searched eight databases and extracted the data on HIV disclosure to partners. Heterogeneity of the data was tested with *I*^2^. Published bias subjectively and objectively analyzed through the funnel plot and Egger's regression test. Subgroup analyses were performed to explore the variation in the proportion by sexual partnership types (unclassified, regular, casual sexual partners), whether being men who have sex with men (MSM), and when to diagnose. The sources of heterogeneity were analyzed. Sensitivity analysis was carried out to evaluate the stability of the results.

**Results:**

Out of 3,698 studies, 44 were included in the review; 11 targeted on MSM. The pooled proportion of HIV disclosure to sexual partners was 65% (95% CI: 56%−75%; 34 studies). Sub-group analyses indicated the proportions of HIV disclosure to regular, casual and unclassified sexual partners were 63% (95% CI: 45%−81%; 31 studies), 20% (95% CI: 8%−33%; nine studies), and 66% (95% CI: 59%−73%; 14 studies), respectively. Fifty-seven percent (95% CI: 45%−69%; three studies) disclosed on the day of diagnosis, 62% (95% CI: 42%−82%; four studies) disclosed within 1 month, and 39% (95% CI: 2%−77%; four studies) disclosed 1 month later. Among MSM, the disclosure to regular male partners, regular female sexual partners, spouses, and casual partner were 47% (95% CI: 29%−65%; six studies), 49% (95% CI: 33%−65%; three studies), 48% (95% CI: 18%−78%; seven studies), and 34% (95% CI: 19%−49%; four studies), respectively.

**Conclusions:**

The disclosure prevalence of people diagnosed with HIV to sexual partners still need improving in China, and it varies among partner types, key populations, and time being diagnosed. HIV disclosure strategies and procedures need to be developed more detailed and tailored based on the pain points of disclosure status, so as to ultimately prevent HIV transmission through sexual contact.

**Systematic review registration:**

https://www.crd.york.ac.uk/prospero/display_record.php?ID=CRD42022291631, identifier: CRD42022291631.

## Introduction

HIV epidemic is a severe global public health problem (1). Since 1981, 79.3 million people have been diagnosed with HIV (PDWH), and 36.3 million have died of AIDS-related illnesses (2). By the end of 2021, 1.14 million PDWH were reported surviving in China (3). Of the newly reported cases in 2021, 97% were self-reported getting infected through sexual contact, and among them, 26% were through male-to-male sexual contact (3). Fear of negative consequences has been shown to be the main barrier for partner disclosure, which includes discrimination, violence, refusal of sex and divorce (4–6). Thus, they may conceal their HIV condition to partners, which might increase HIV transmission through sexual contact (7).

HIV partner disclosure has been advocated by World Health Organization (WHO) to reduce HIV transmission through sexual contact, especially for HIV key populations, men who have sex with men (MSM), female sex workers, drug users, and transgender people (8, 9). The HIV disclosure rate varied between countries and was lowest in developing countries (16.7%−86%) (10). In China, the disclosure of HIV is usually based on PDWH's own willingness (11). Despite more open policies about partner notification strategy for sexually transmitted diseases (12, 13), promotion of safer sex (14–16), and increased numbers in HIV testing, the proportion of HIV partner disclosure in China was very inconsistent (17). Literatures recorded the disclosure rate in China to be between 11.4% (18) and 90.2% (19), which could not reflect the overall disclosure situation in China.

We systematically searched eight databases (PubMed, Cochrane Library, Embase, Web of Science, China National Knowledge Internet, Wan Fang, Sino Med, and VIP data) using the terms: “HIV,” “sexual partner,” and “disclosure” and found 17 published systematic reviews. They focused on influencing factors of HIV disclosure (14, 20–22), disclosure policies and their effectiveness (13, 23, 24), interventions for HIV disclosure (25, 26), evaluation of self-report disclosure tools (27), and the HIV disclosure among specific populations such as adolescents or immigrants (28–30). Four reviews synthesized the proportions of HIV partner disclosure among Ethiopian adults with PDWH (31–34). One (31) reported 76.03% of pooled disclosure rate (18 studies, 8,009 participants), while the other (32) reported 73% of disclosure rate (12 studies, 4,528 participants). Two other reviews (33, 34) reported 74.63% (22 studies, 8,873 participants) and 75.95% (18 studies, 7,084 participants) disclosure proportions, respectively. However, there is still a lack of knowledge about the special characteristics of HIV disclosure to sexual partners (31–34). The quality of the reviews was also low, resulting in high publication bias (32, 34). Considering that sexual contact has become the main transmission route of the increasing global HIV epidemic, disclosing HIV to sexual partners could be an effective strategy in preventing secondary HIV transmission from HIV high-risk populations to the general population (35, 36). However, China still uses non-systematically evaluation of the HIV partner disclosure, which makes the development of HIV disclosure promotion programs challenging (37). Therefore, given the high prevalence of HIV sexual transmission in China, the large number of PDWH and the treatment burden, it is important to focus on Chinese literature about HIV disclosure to sexual partners (38, 39). Considering some studies conducted in China were published in Chinese journals, which were not indexed in English databases, we selected four Chinese databases (China National Knowledge Internet, Wan Fang, Sino Med, and VIP data), which would cover almost all the studies published in Chinese.

We aimed to conduct a systematic review and meta-analysis to determine the pooled proportions of HIV disclosure to sexual partners in China. We defined HIV disclosure as PDWH notifying partners voluntarily by themselves, including active notification (i.e., spontaneous notification after diagnosis) or passive notification (after being advised by the health care professional, the PDWH choose to disclose on his own) (40). We also combined HIV disclosure proportions under different situations, such as disclosure among MSM, to different sexual partnership types, and when to disclose after diagnosis. We hope that the knowledge gained by this review could provide references to enrich the partner disclosure policies for Chinese policymakers, guide the development of targeted partner disclosure promotion interventions, and assist the achievement of the ultimate goal of ending AIDS in 2030 (41).

## Methods

This review synthesized the disclosure proportion using the JBI methodology for single prevalence or incidence systematic reviews (42). The protocol was registered in the PROSPERO database (CRD42022291631). The review was reported according to the Preferred Reporting Items for Systematic Reviews and Meta-Analyses (PRISMA) guidelines (43).

### Data source and search strategy

A comprehensive search was carried out using eight electronic databases, including four Chinese (the China National Knowledge Internet, Wan Fang, Sino Med, and VIP data) and four international databases (PubMed, Cochrane Library, Embase, and Web of Science). These databases were selected as they cover as much of the literature we need as possible (44). The search was limited to all primary studies published from 1981 until the search date. Gray literature, including conference abstracts, graduate dissertations, and unpublished articles were screened using Google Scholar. We contacted the authors if there was any doubt about the data or if further details were needed. In addition, we searched references of included studies for potentially eligible studies (45).

The main search terms and phrases were “HIV,” “AIDS,” “HIV disclosure,” “sexual partner,” “reveal,” “partner notification,” “China,” and “Chinese.” For example, Boolean search using AND, OR were used in search strategy in PubMed as follows: [HIV [MeSH Terms] OR HIV infections [MeSH Terms] OR Acquired Immunodeficiency Syndrome [MeSH Terms] OR Human immunodeficiency virus OR HIV infections OR AIDS OR Acquired Immunodeficiency Syndrome (AIDS)] AND [Truth Disclosure [MeSH Terms] OR Self Disclosure [MeSH Terms] OR Disclosure [MeSH Terms] OR HIV Disclosure OR HIV serostatus disclosure OR partner disclosure OR (disclos^*^) OR (expos^*^) OR (reveal^*^) OR partner notification] AND (China OR Chinese). We adjusted the retrieval formula on this basis according to different databases. All citations were imported into Endnote 20.0 to find and remove duplicates. Detailed search formulas for each database were provided in the Supplementary Table S1.

### Eligibility criteria

We applied the following inclusion criteria: (1) studies reported HIV disclosure of PDWH (≥18 years old) (46) who have at least one sexual partner; (2) disclosing to one or more partners (regardless of regular or casual sexual partners) counts as eligible; (3) all observational (cross-sectional, cohort, and case-control) studies assessed HIV disclosure in China. Baseline data from randomized controlled studies, mixed studies, and intervention studies would also be used; (4) studies published after 1981, when the first five AIDS cases were reported in the world (47). The search was restricted to English and Chinese language.

The exclusion criteria were: (1) studies including PDWH who did not explicitly report having a sexual partner would be excluded; (2) reported data could not extract the disclosure rate; (3) studies were qualitative, reviews, systematic reviews, or meta-analyses.

### Selection of studies

Two reviewers (WP and XS) independently searched the literature, browsed through the titles and abstracts, and finally screened the full text that met the inclusion criteria (48). Discrepancies were resolved by consultation or discussion with a third reviewer (XL). For studies excluded, we recorded the reasons for rejection to ensure a transparent and open selection process. For multiple articles with the same study data, we only retained one published article that met the inclusion criteria, especially which one had extractable data, and combined the same data as one.

### Data extraction

Once the eligible studies have been identified, two reviewers extracted data on the variables (including author, year, province, study design, population, sample size, types of sexual partners reported, and HIV disclosure events; Table 1). Data extraction has been done independently by two reviewers (WP and XS), and followed by comparison to ensure data accuracy; differences were resolved through joint discussion by team members (48).

**Table 1:** Characteristics and quality of included studies.

**No**.	**Authors year**	**Province**	**Study design**	**Study population**	Sample size^*^(male/female)	**Outcomes**		**Quality score**
						**Type of sexual partner**	HIV disclosure events^**^	
1	Wu 2021	Chongqing	Cohort study	PDWH: unclassified	312 (207/105)	Spouse	156/175	5
2	Qi 2012	Yunnan	Cross-sectional study	PDWH: unclassified	300 (186/114)	Spouse	101/300	8
3	Zhao 2016	Jiangsu	Cohort study	PDWH: unclassified	152 (142/10)	Spouse	75/152	7
4	Ni 2011	Xinjiang	Cross-sectional study	PDWH: unclassified	3,071 (1,900/1,171)	Unclassified	2,010/2,467	7
5	Xue 2011	Xinjiang	Cross-sectional study	PDWH: unclassified	257 (217/40)	Unclassified	139/154	6
6	Zhuo 2020	Sichuan	Cross-sectional study	PDWH: unclassified	850 (670/180)	Spouse	735/850	8
7	Huang 2018	Shenyang	Cross-sectional study	PDWH:only MSM	524 (524/0)	Spouse	72/115	6
8	Wang M 2013	Shanghai	Cross-sectional study	PDWH:only MSM	200 (200/0)	Regular male partners	78/200	8
9	Hu 2017	Shanxi	Cross-sectional study	PDWH: unclassified	223 (212/11)	Spouse	75/89	8
10	Yu 2017	Yunnan	Case-control study	PDWH: unclassified	223 (–/–)	Spouse	91/223	8
11	Shan 2010	Yunnan	Cross-sectional study	PDWH: unclassified	497 (250/247)	Regular sexual partner	307/389	8
						Casual sexual partner	1/16	
12	Chen 2019	Unclear	Cross-sectional study	PDWH: unclassified	243 (218/25)	Unclassified	113/144	8
13	Hu 2014	Guangxi	Cross-sectional study	PDWH: unclassified	425 (294/131)	Regular sexual partner	245/425	8
14	Gao 2010	Yunnan	Cross-sectional study	PDWH: unclassified	305 (165/140)	Regular sexual partner	128/283	8
						Casual sexual partner	3/160	
15	Asimuguli 2021	Xinjiang	Cross-sectional study	PDWH: unclassified	201 (130/71)	Unclassified	131/201	8
16	Wang Q 2013	Henan	Cross-sectional study	PDWH: unclassified	557 (210/347)	Spouse	203/557	5
17	Lan 2020	Guangxi	Cross-sectional study	PDWH: only MSM	91 (91/0)	Regular female sexual partner	28/91	8
						Regular male partners	24/91	
18	Chen 2010	Unclear	Cross-sectional study	PDWH: unclassified	23 (14/9)	Spouse	19/23	5
19	Yang 2015	Guangxi	Case-control study	PDWH: unclassified	397 (299/98)	Spouse	388/397	8
20	Qin 2021	Anhui	Cross-sectional study	PDWH: unclassified	217 (170/47)	Spouse	163/217	6
21	Mi 2010	Sichuan	Cross-sectional study	PDWH: only MSM	202 (202/0)	Regular sexual partner	48/109	6
						Casual sexual partner	15/106	
22	Liu 2013	Hunan	Cross-sectional study	PDWH: unclassified	262 (207/55)	Unclassified	137/262	8
23	He 2021	Beijing	Cross-sectional study	PDWH: unclassified	200 (200/0)	Unclassified	118/188	8
24	Liu 2011	Shandong	Cross-sectional study	PDWH: unclassified	213 (–/–)	Spouse	102/117	7
25	Li 2021	Sichuan	Cross-sectional study	PDWH: unclassified	283 (212/71)	Unclassified	236/283	8
26	Yang 2005	Beijing, Guangdong	Cross-sectional study	PDWH: unclassified	214 (148/66)	Unclassified	119/214	6
27	Xu 2011	Henan, Zhejiang, Gansu, Yunnan	Mixed study	PDWH: unclassified	481 (331/150)	Spouse	440/481	6
28	Zhou 2014	Jiangsu	Cross-sectional study	PDWH: only MSM	164 (164/0)	Spouse	109/164	8
29	Yang 2011	Hubei	Cross-sectional study	PDWH: only MSM	100 (100/0)	Regular sexual partner	19/100	5
30	Yang 2018	Jiangsu	Cross-sectional study	PDWH: unclassified	466 (443/23)	Regular male partners	115/150	8
						Spouse	128/180	
31	Jin 2017	Guangdong	Cross-sectional	PDWH: only MSM	340 (340/0)	Unclassified	162/253	8
			study			Regular sexual partner	135/200	
						Spouse	12/31	
						Casual sexual partner	67/148	
32	Liu 2017	Shanghai, Sichuan	Cross-sectional study	PDWH: only MSM	308 (308/0)	Regular sexual partner	174/274	8
						Spouse	38/83	
						Casual sexual partner	62/210	
33	Xiao 2015	Guangxi	Cross-sectional study	PDWH: unclassified	2,987 (–/–)	Unclassified	125/1,093	6
34	Mao 2018	Guangxi	Cross-sectional study	PDWH: unclassified	1,254 (742/512)	Regular sexual partner	851/1,254	7
35	Xiao 2018	Hunan	Cross-sectional study	PDWH: unclassified	184 (133/51)	Unclassified	68/104	8
36	Yan 2019	Hebei, Sichuan, Jiangsu	Cross-sectional study	PDWH: only MSM	432 (432/0)	Regular female sexual partner	215/432	8
37	Yan 2021	Guangdong	Cross-sectional study	PDWH: only MSM	944 (944/0)	Regular sexual partner	300/461	6
						Casual sexual partner	194/416	
38	Ding 2011	Xinjiang, Yunnan	Cross-sectional study	PDWH: unclassified	88 (53/35)	Unclassified	54/88	8
39	Wang 2010	Guangxi, Yunnan	Cross-sectional study	PDWH: unclassified	946 (494/452)	Regular sexual partner	620/654	7
						Casual sexual partner	7/54	
						Unclassified	625/693	
40	Qiao 2016	Guangxi	Cross-sectional study	PDWH: unclassified	791 (420/371)	Unclassified	405/791	7
41	Zang 2015	Guangxi	Cross-sectional study	PDWH: unclassified	147 (103/44)	spouse	88/92	6
42	Chen 2014	Guangdong, Chongqing, Sichuan	Cross-sectional study	PDWH: only MSM	541 (541/0)	Regular male partners	263/423	8
						Spouse	19/423	
43	Chen 2013	Gansu	Cross-sectional study	PDWH: unclassified	232 (206/26)	Regular sexual partner	94/148	8
						Casual sexual partner	6/114	
44	Zhang 2009	Yunnan, Guangxi	Mixed study	PDWH: unclassified	974 (553/421)	Regular sexual partner	516/549	8
						Casual sexual partner	11/54	

PDWH unclassified: The study participants included were people diagnosed with HIV, and no distinction has been made between special groups such as men who have sex with men (MSM); PDWH only MSM: The study participants included were MSM populations diagnosed with HIV.

* Sample size means the number of people living with HIV included in the study.

** HIV disclosure events were calculated by the formula: The number of people who disclosed HIV to (some type) of sexual partners/the number of people who had (certain types of partners).

### Evaluation of study quality

The studies included were assessed by two reviewers (WP and QZ) independently, using the Joanna Briggs Institute (JBI) Critical Appraisal Checklist for Analytical Cross-Sectional Studies (49). Since we used baseline data only from intervention studies, cohort studies, and mixed studies, we also evaluated them using the checklist. Each item rated “yes” referring value “1” was summed giving a range of a possible total score between 0 and 8 on the checklist. Based on Zhang's method (50), we classified below 3 as low quality, 4–7 as medium quality, and above 7 as high quality (Table 1). Discrepancies were resolved by consultation or discussion with a third reviewer (CZ).

### Statistical analysis

Extracted data was exported to Stata 16.0 for meta-analysis. The statistical heterogeneity of the pooled rate was assessed according to *I*^2^ with a *p*-value. *p*-value < 0.05 indicated heterogeneity existence. The *I*^2^ value presented low, medium and high heterogeneity by the cut of 25, 50 and 75% values (51). Since high heterogeneity was shown in the final data, we employed the random effects model to estimate the pooled proportion and produce 95% confidence intervals (CI) (52, 53).

We checked the publication bias using a funnel plot, by judging the symmetry of the figure. We also conducted an Egger's regression test, which would be suggestive of the significant absence of publication bias if the *p*-value is more than 0.05 (54). In addition, subgroup analyses were undertaken based on the different types of sexual partners (unclassified, regular, and casual sexual partners) among the common population and MSM, and when to disclose (on the day ofdiagnosis, within 1 month, and 1 month later after diagnosis). Sensitivity analysis was conducted to assess the stability of the pooled proportion by eliminating one study in each turn (55).

## Results

### Study characteristics

**Figure 1:**
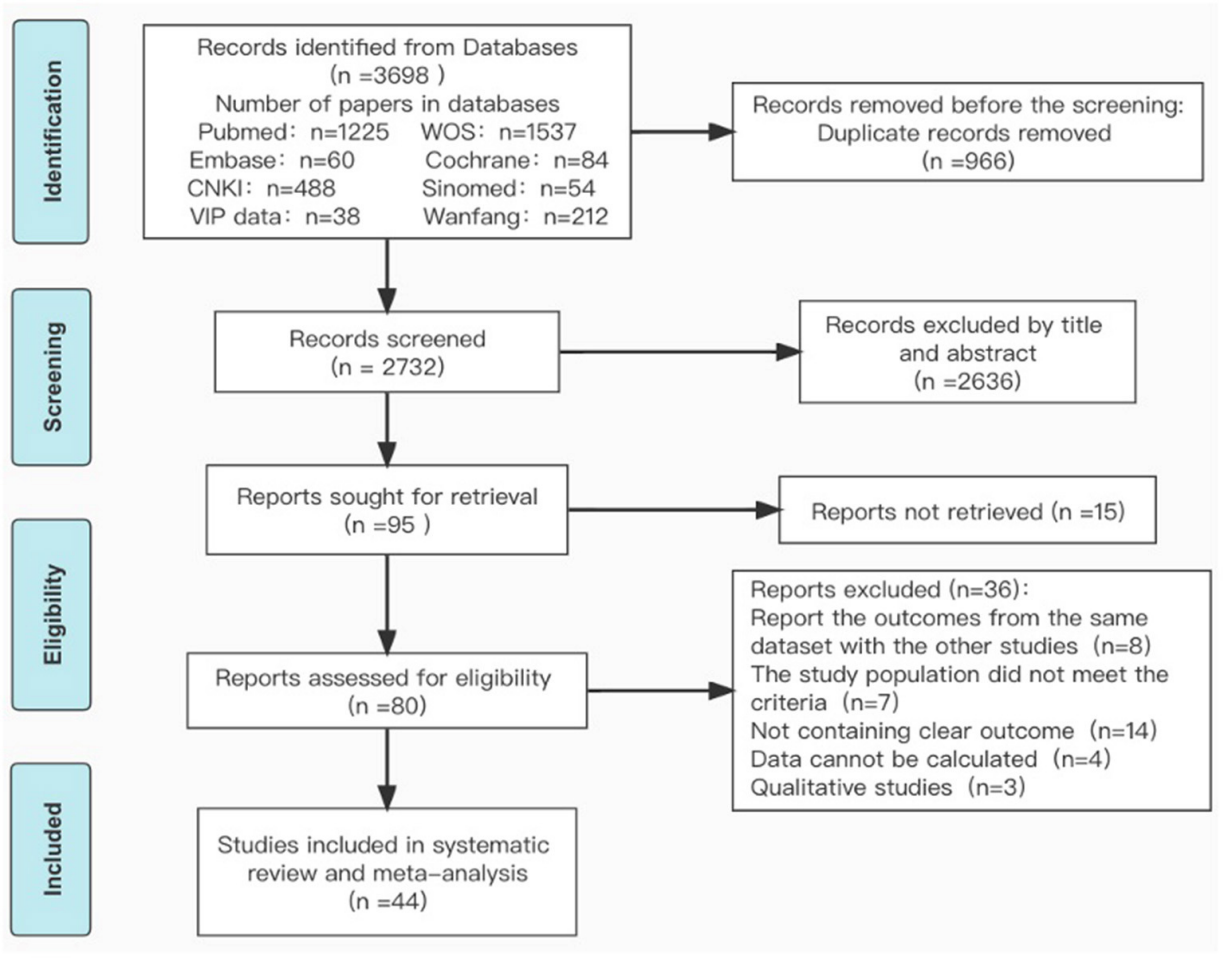
Flow chart of literature search and selection procedures and outcomes.

Searches of the literature were up to November 23, 2021. We retrieved 3,698 studies from eight databases. Among these, 3,644 articles were excluded and 44 studies were selected for data synthesis. The selection process and reasons for exclusion were illustrated in Figure 1.

All included 44 studies (19, 56–62) were published between 2005 and 2021. The study regions covered 55.88% (19/34) of provinces in China (Table 1). Thirty-five studies (19, 56–62) were published in Chinese and the remaining nine studies were published in English (18, 63–70). Except for two case-control (71, 72), two cohort studies (56, 58), and two mixed studies (73, 74), 38 studies were cross-sectional studies (19, 57, 60, 62, 66–69, 75–83). A total of 21,821 PDWHs were included in the review, of which 3,846 (17.6%) were MSM. The male to female ratio was 2.66 in the pooled study population. In addition, among the included MSM, 1,223 (31.8%) had regular male partners, 984 (25.6%) had regular female partners, 1,011 (26.3%) had spouses, and 880 (22.9%) had casual sexual partners.

### Quality assessment

The average score for the quality appraisal for the 44 studies was 7.2. Of these, 25 studies (57, 60, 62, 71, 77, 81, 84, 85) were high-quality (56.8%) and the rest of the studies were on moderate quality (43.2%); no low-quality studies were identified (Table 1, Supplementary Table S2).

### The general proportion of HIV partner disclosure

**Figure 2:**
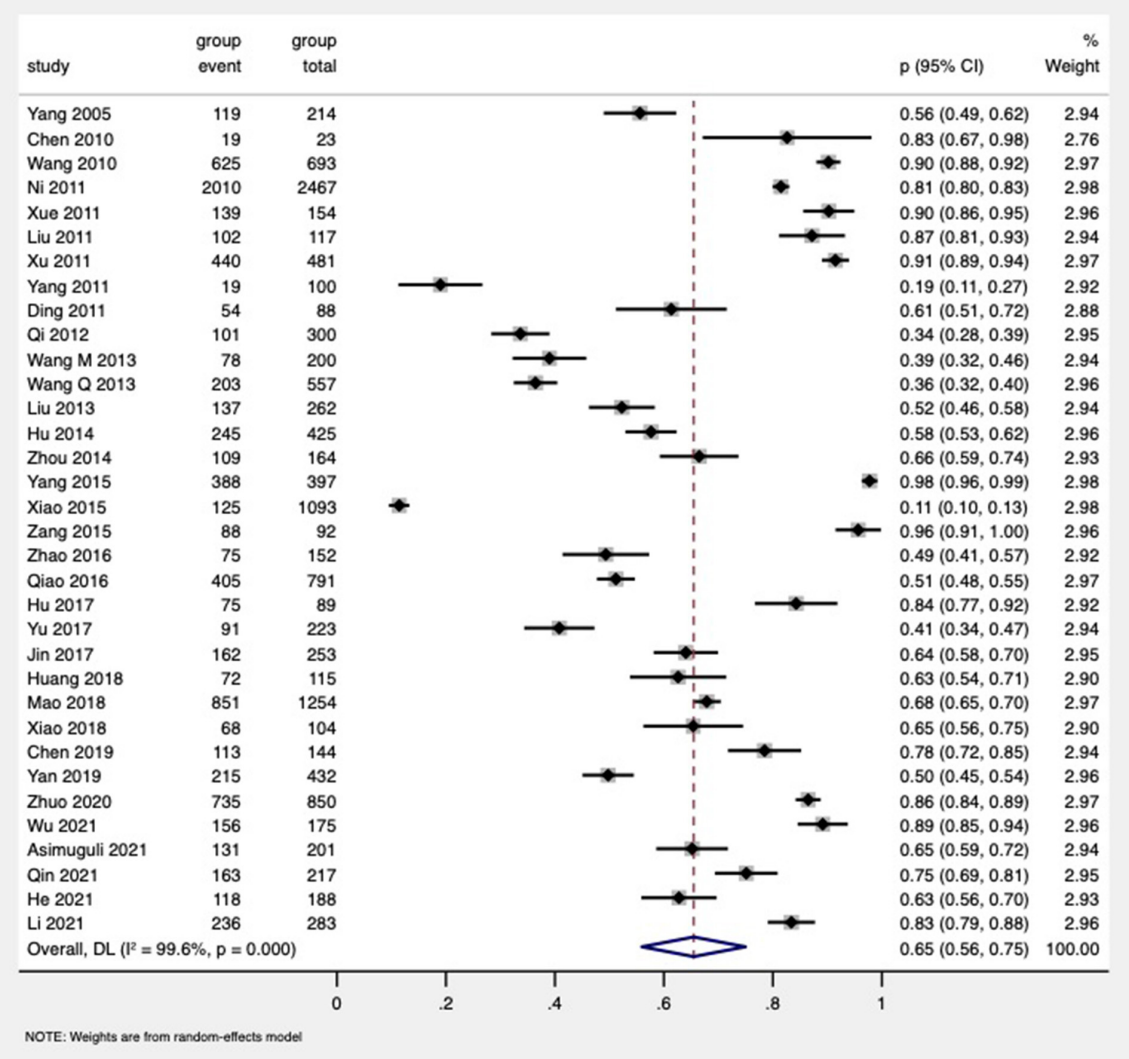
Forest of the pooled proportion of HIV disclosure to sexual partners of PDWH in China.

The proportion of HIV disclosure to sexual partners ranged from 11 to 98% (18, 72) reported by 34 studies (19, 56–62), and the pooled proportion was found to be 65% (95% CI: 56%−75%), with a high level of heterogeneity (*I*^2^ = 99.6%; *p* < 0.001) (Figure 2). We conducted subgroup analyses to explore the variation of partner disclosure rates, which were reported below (Supplementary Table S3).

#### The proportion of partner disclosure to different types of sexual partners

**Figure 3:**
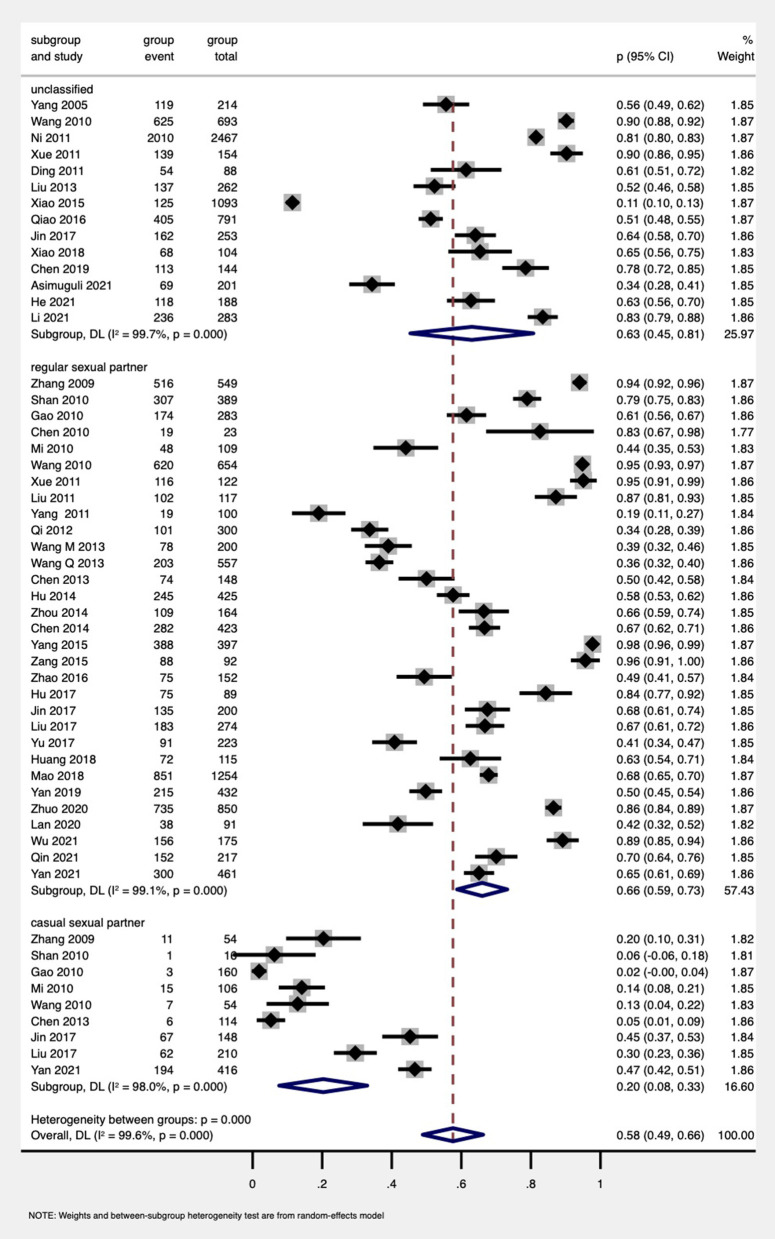
Forest plot of subgroup analysis by types of sexual partners among PDWH.

Among all PDWH (including MSM) (19, 56–62), the subgroup analysis indicated that the proportion of HIV disclosure to unclassified types of sexual partners was similar (63%, 95% CI: 45%−81%, 14 studies) to regular sexual partners (66%, 95% CI: 59%−73%, 31 studies), and both of them were far higher than the disclosure to casual sexual partners (20%, 95% CI: 8%−33%, nine studies; Figure 3).

#### The proportion of partner disclosure at different times

**Figure 4:**
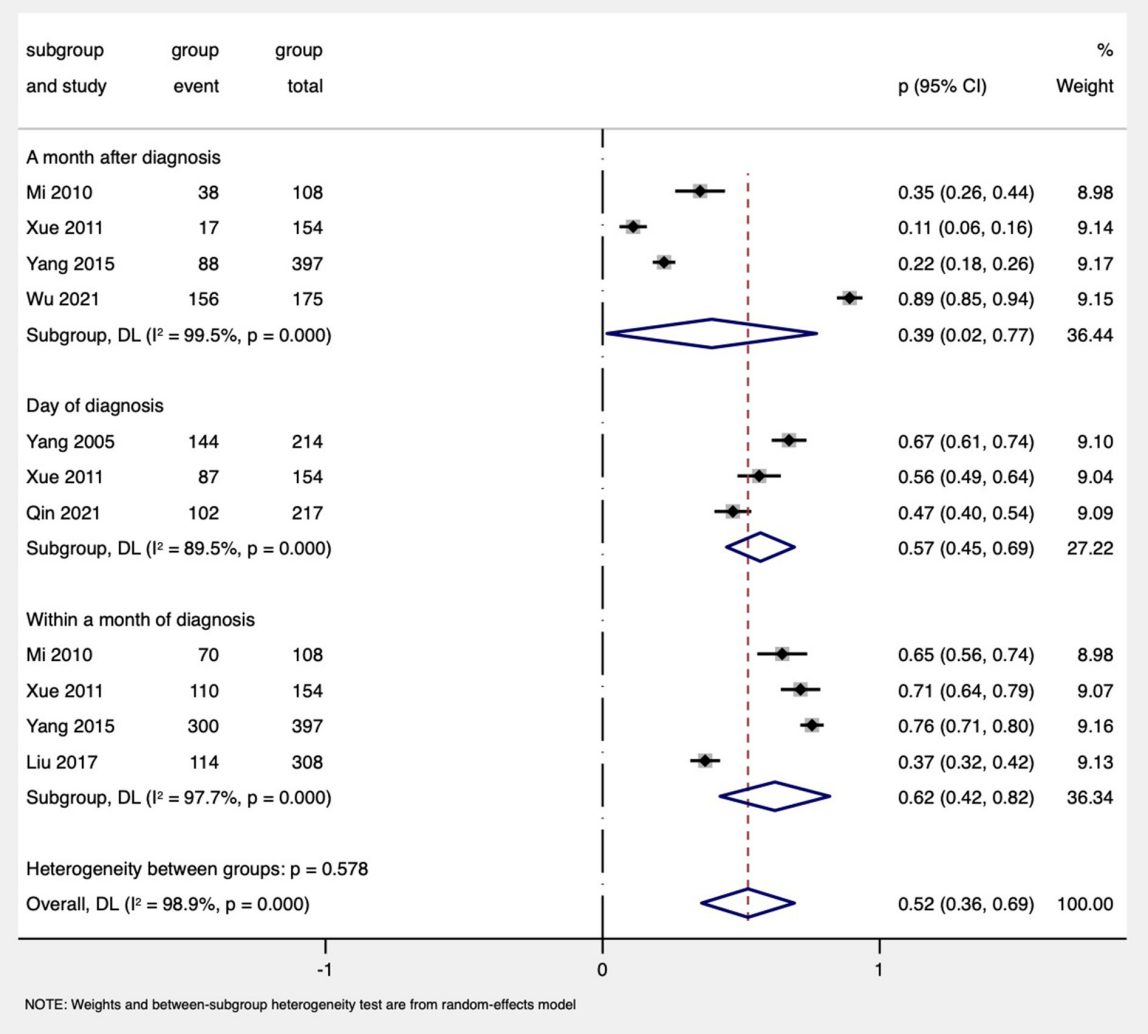
Forest plot of subgroup analysis by disclosure at different diagnosis times among PDWH.

The subgroup analysis based on seven studies was performed (19, 56, 72, 79, 86–88). The analysis suggested that the highest disclosure proportion was within 1 month after diagnosis (62%, 95% CI: 42%−82%, four studies), followed by on the day of diagnosis (57%, 95% CI: 45%−69%, three studies), and the lowest disclosure rate was at 1 month later after diagnosis (39%, 95% CI: 2%−77%, four studies; Figure 4).

#### The proportion of HIV disclosure to different types of sexual partners among MSM

**Figure 5:**
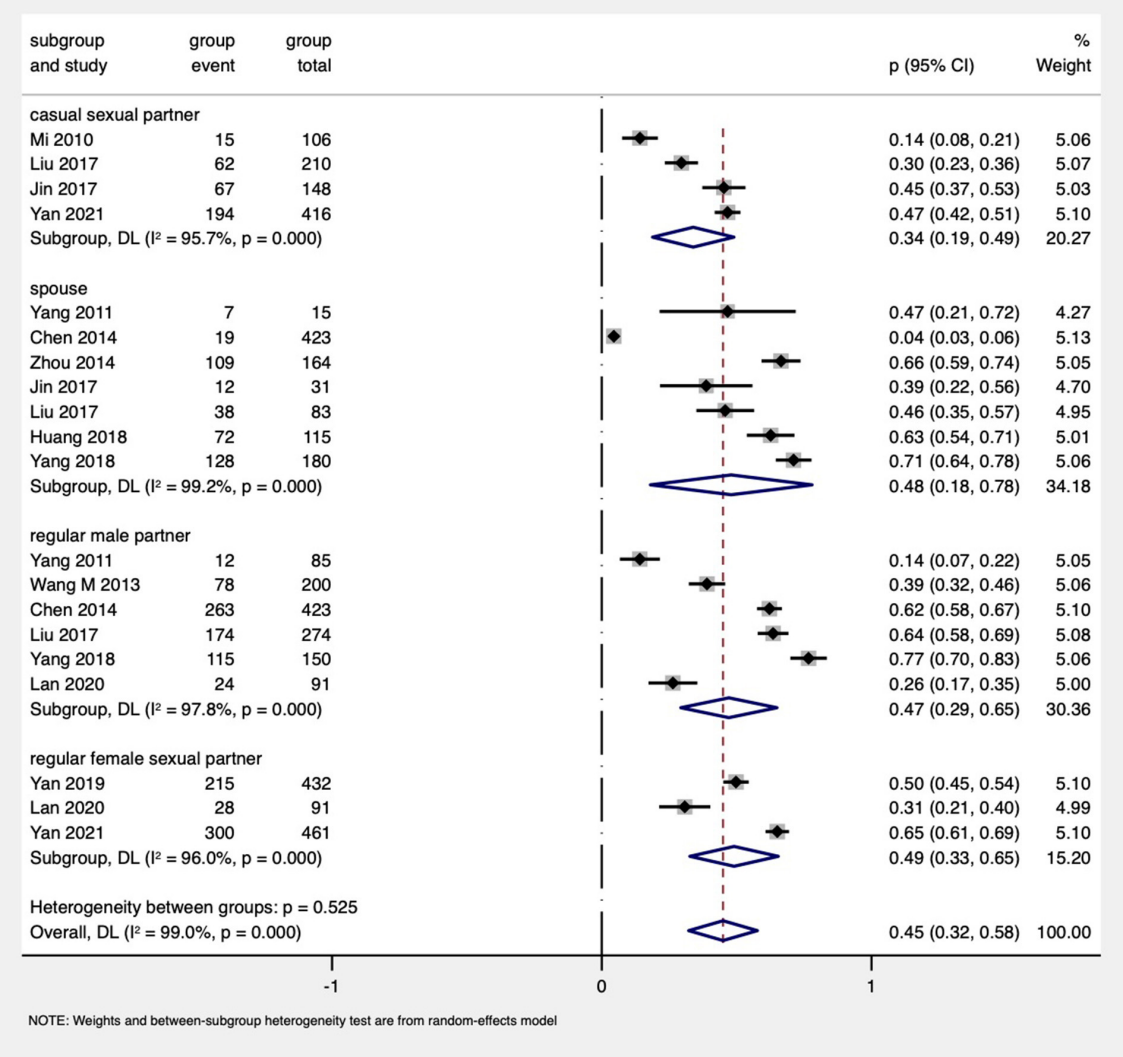
Forest plot of subgroup analysis by types of sexual partners among MSM PDWH.

As for MSM, 11 studies (61, 62, 78, 81, 86, 89–91) reported the disclosure rate to regular partners, and four studies (66, 86, 88, 91) reported the disclosure to casual partners. The proportions of disclosure to regular male partners, regular female sexual partners, and spouses among the MSM were 47% (95% CI: 29%−65%, six studies), 49% (95% CI: 33%−65%, three studies), and 48% (95% CI: 18%−78%, seven studies), respectively. MSM had the lowest rate of HIV disclosure to casual partners (34%, 95% CI: 19%−49%, four studies; Figure 5).

### Risk of bias across studies

The presence of publication bias was evaluated both subjectively and objectively. The funnel plots seemed symmetric (Supplementary Figure S1), and Egger's regression test (*t* = −1.27, *p* = 0.213) also indicated the absence of publication bias.

### Sensitivity analysis

We conducted a sensitivity analysis by removing those four studies (56, 78, 80, 92) where their quality score was the lowest (five score), which may influence the overall rate. We removed one study in each turn, and then removed all these four studies. We recalculated the pooled estimation on the remaining studies. The combined results of overall rate estimates were consistent and without apparent fluctuation, with a narrow range from 0.65 (95% CI: 0.55–0.74) to 0.67 (95% CI: 0.57–0.76), which was similar to the primary results. This analysis confirmed the stability of the pooled results (Supplementary Figure S2).

## Discussion

In this review, the overall prevalence of HIV disclosure to sexual partners among Chinese PDWH was 65%. Our findings support previous studies conducted in Togo (93) and Uganda (94). On the contrary, our HIV disclosure rate was lower than in the United States (95), and some African countries, such as Kenya (96), South Africa (97), Ethiopia (31).

There might be two rationales for the low HIV disclosure rate in China. First, the different regional backgrounds and HIV disclosure policies in the world would affect HIV partner disclosure. For example, the United States has long regarded partner notification as an important strategy for AIDS prevention, which may promote HIV disclosure (98–100). And in several African countries which were severely affected by HIV, laws on HIV disclosure have also been issued (101). Hence, due to the mature legal policies, self-disclosure in these regions was higher. However, at present, China has only a national-level policy of “prevention and control regulation” (102), stating that HIV partner disclosure should be totally voluntary. Just four provinces in China have issued local mandatory policies on spouse notification (103, 104), which have been shown to effectively increase PDWH's activeness of HIV disclosure in these areas (73). Second, HIV-related stigma toward PDWH has been confirmed to be the key barrier for HIV disclosure (105). In China, HIV stigma is higher than in developed countries and some African countries (106, 107). In addition, compared to American and European countries (108–110), China has demonstrated a higher judgmental attitude toward MSM due to the traditional social norm and Confucianism philosophy (111). Our previous study also indicated that married MSM PDWH in China would prefer to disclose HIV status than sexual orientation, however, disclosing HIV status to sexual partners will increase the risk of sexual orientation exposure, which make them conceal their HIV status (112). That is why the regular partner disclosure rate was very low among MSM (47%−49%) as shown by our synthesized data.

Our results indicated that the disclosure proportions varied according to different sexual partnerships being highest in regular partners and lowest in casual partners. This review showed that PDWH tend to disclose to regular partners (66%) rather than casual partners (20%), which was similar to the studies conducted in both developing and developed countries like Africa and America (95, 113, 114). Regular sexual partners (115) were defined as whom they had stable sex relationships for more than 3 months, including legally married spouses (116). As an intimate relationship, regular partners can provide PDWH with emotional support, treatment advice, and coping strategies for HIV-related stigma (117). However, casual partners are mainly acquainted in the process of one-night stands or commercial sex (118), thus the relationships are often built on sexual stimulation or an exchange of money, which are unstable or weaker than that with regular partners. Besides, usually, it is not easy to find the causal sexual partners again to notify the status (119), and they also have a low sense of responsibility to disclose to such partners (120, 121). In terms of MSM, our synthesized result also showed they were reluctant to disclose to causal sexual partners (34%).

The synthesized results also indicated that HIV disclosure varied at different times after diagnosis, which was supported by the study in Tanzania (122). In this review, PDWH would prefer to notify partners within 1 month of diagnosis if they decided to disclose; this finding support previous results in Nigeria (123). Since an HIV-positive diagnosis is a stressful event for patients, they tend to take an active approach to the disease response, hoping to alleviate fear and shame by disclosing as soon as possible (122, 124). In addition, if they did not disclose within 1 month's diagnosis, along with the improvement of CD4 counts and reduced viral load, they would become more reluctant to disclose, as indicated by a study conducted in Kenya (124), which is also supported by our synthesized results.

This systematic review and meta-analysis have a couple of limitations, which should be carefully considered. First, there may be a selection bias due to the studies included in this review being limited to Chinese and English, which could not represent articles published in other languages. Second, there may exist a reporting bias. In the included studies, the measures of HIV disclosure were mostly self-reported by participants, which may lead to information bias. Third, although we have conducted subgroup and sensitivity analyses, there was still large heterogeneity in the results. It might be due to the large variation of the sample size in the included studies, and the wide coverage of the study settings, which included 19 provinces in China, where the partner disclosure policies were significantly different as discussed above. In addition, some studies included only MSM, which may also lead to a large heterogeneity in the final pooled disclosure proportion. Finally, the literature retrieval was not completed on the same day, which may result in potentially inconsistent query answers from the database itself (125).

This systematic review has several implications for future studies and practices. First, it provides evidence for policymakers to consider how to systematically promote HIV partner disclosure. Potential ethical issues should be considered during the policy development. Second, timely HIV disclosure can promote HIV testing of partners, avoid high-risk sexual behavior, facilitate condom use, and reduce HIV transmission between partners (126, 127). Therefore, patients should be encouraged to disclose to their partners in the early stage of diagnosis. Third, intervention studies could be designed in future studies by targeting especially the low disclosure rate population of MSM, and the causal sexual partners of HIV infected, so as to prevent HIV transmission through HIV key populations to the general population.

## Conclusions

The pooled HIV disclosure to sexual partners in China was 65%. The lowest disclosure was to casual partners, and the disclosure significantly decreased after 1 month of diagnosis, it will raise a concern in the development of HIV disclosure policy. Therefore, the government or relevant health departments need to develop more detailed HIV disclosure strategies, especially for patients with multiple sex partners, so as to ultimately prevent HIV transmission through sexual contact, and achieve the “95–95–95” target in 2030 (128).

## Data availability statement

The raw data supporting the conclusions of this article will be made available by the authors, without undue reservation.

## Author contributions

WP and XS conducted the literature search, evaluated the study quality, and extracted the data. WP and QZ analyzed the data by Stata software. MV and XL supervised the draft writing. WP wrote the main manuscript text. All the authors determined the methodology and reviewed and edited the manuscript.

## Funding

This study was funded by the National Natural Science Foundation of China (XL; 72074226; https://isisn.nsfc.gov.cn/egrantweb/). The funder had no role in the design of the study and collection, analysis, and interpretation of data and the manuscript writing.

## Conflict of interest

The authors declare that the research was conducted in the absence of any commercial or financial relationships that could be construed as a potential conflict of interest.

## Publisher's note

All claims expressed in this article are solely those of the authors and do not necessarily represent those of their affiliated organizations, or those of the publisher, the editors and the reviewers. Any product that may be evaluated in this article, or claim that may be made by its manufacturer, is not guaranteed or endorsed by the publisher.

## Supplementary material

Supplementary Table S1 The search terms used in the databases and the number of results.

Supplementary Table S2 Assessment of methodological quality of analytical cross-sectional studies (*n* = 44).

Supplementary Table S3 Subgroup analyses of HIV disclosure to sexual partners.

Supplementary Figure S1 Funnel plot of HIV disclosure to sexual partners among PDWH.

Supplementary Figure S2 Sensitivity analysis for the pooled proportion of HIV disclosure to sexual partners.

## References

[1] Gazzard B. AIDS. Baillieres Clin Gastroenterol. (1987) 1:567–87. 10.1016/0950-3528(87)90048-0 3322433

[2] UNAIDS. Fact sheet: World AIDS day 2020. Global HIV statistics. Geneva: UNAIDS (2020).

[3] CCTV News: Low pop level! Our country reports 1,.14 million cases of HIV infection. (2021). Available online at: https://china.huanqiu.com/article/45nyYDqU4MN (accessed June 28, 2022).

[4] Gostin LO Feldblum C Webber DW. Disability discrimination in America: HIV/AIDS and other health conditions. JAMA. (1999) 281:745–52. 10.1001/jama.281.8.745 10052447

[5] Pakenham K Rinaldis M. Development of the HIV/AIDS stress scale. Psychol Health. (2002) 17:203–19. 10.1037/t07009-00027043134

[6] Jaspal R. Stigma and HIV Concealment motivation among gay men living with HIV in Finland. J Homosex. (2022) 69:699–715. 10.1080/00918369.2020.1851958 33320069

[7] Suarez TP Kelly JA Pinkerton SD Stevenson YL Hayat M Smith MD . Influence of a partner's HIV serostatus, use of highly active antiretroviral therapy, and viral load on perceptions of sexual risk behavior in a community sample of men who have sex with men. J Acquir Immune Defic Syndr. (2001) 28:471–7. 10.1097/00042560-200112150-00011 11744837

[8] Consolidated Guidelines on HIV Testing Services. Geneva: World Health Organization (2015). Available online at: https://www.who.int/publications/i/item/978-92-4-1 (accessed June 9, 2022).

[9] WHO/UNAIDS. WHO/UNAIDS. Guidelines for Conducting HIV Sentinel Serosurveys among Pregnant Women and Other Groups. Geneva: WHO/UNAIDS (2003) [updated 2003-12-01]. Available online at: https://data.unaids.org/publications/irc-pub06/jc954-anc-serosurveys_guidelines_en.pdf (accessed June 28, 2022).

[10] Medley A Maman S. Gender Dimensions of HIV Status Disclosure to Sexual Partners: Rates, Barrier and Outcomes. Geneva: World Health Organization.

[11] WHO Guidelines Approved by the Guidelines Review Committee. Guidelines on HIV Self-Testing and Partner Notification: Supplement to Consolidated Guidelines on HIV Testing Services. Geneva: World Health Organization (2016). 27977094

[12] Mathews C Coetzee N Zwarenstein M Lombard C Guttmacher S Oxman A . Strategies for partner notification for sexually transmitted diseases. Cochrane Database Syst Rev. (2001) 4:CD002843. 10.1002/14651858.CD002843 11687164

[13] Wang AL Peng RR Tucker JD Cohen MS Chen XS. Partner notification uptake for sexually transmitted infections in China: a systematic literature review. Sex Transm Infect. (2012) 88:386–93. 10.1136/sextrans-2011-050275 22427489 PMC3667599

[14] Smith R Rossetto K Peterson BL. A meta-analysis of disclosure of one's HIV-positive status, stigma and social support. AIDS Care. (2008) 20:1266–75. 10.1080/09540120801926977 18608080

[15] Dalal S Johnson C Fonner V Kennedy CE Siegfried N Figueroa C . Improving HIV test uptake and case finding with assisted partner notification services. AIDS. (2017) 31:1867–76. 10.1097/QAD.0000000000001555 28590326 PMC5538304

[16] Katz BP Caine VA Jones RB. Evaluation of field follow-up in a sexually transmitted disease clinic for patients at risk for infection with *Neisseria gonorrhoeae* and *Chlamydia trachomatis*. Sex Transm Dis. (1992) 19:99–104. 10.1097/00007435-199219020-00007 1595019

[17] Lu Z Zhenzhen L Honghong W. Research progress on influencing factors of partner notification in HIV infection/AIDS patients. Chin Nurs Res. (2014) 28:4106–8. 10.3969/j.issn.10096493.2014.33.004

[18] Xiao Z Li X Qiao S Zhou Y Shen Z Tang Z. Using communication privacy management theory to examine HIV disclosure to sexual partners/spouses among PLHIV in Guangxi. AIDS Care. (2015) 27:73–82. 10.1080/09540121.2015.1055229 26616128 PMC4699476

[19] Guo-fang X Fan L Rui-lan L Yu-juan Z Jin-bao L Yong-bo P. The analysis of HIV infections' inform intention of MMT personnel in Urumqi. J China Tradit Chin Med Inf. (2011) 3:469–70. [in Chinese].

[20] Li H Li X Zhang L Chow E. Effects of multiple types of stigma on the probability of HIV disclosure to sex partners: a systematic review. Sex Health. (2016) 13:516–29. 10.1071/SH16089 27491829

[21] Whembolua GL Conserve DF Thomas K Tshiswaka DI Handler L. HIV serostatus disclosure in the Democratic Republic of the Congo: a systematic review. AIDS Care. (2019) 31:489–93. 10.1080/09540121.2018.1510103 30111174

[22] Adeoye-Agboola DI Evans H Hewson D Pappas Y. Factors influencing HIV disclosure among people living with HIV/AIDS in Nigeria: a systematic review using narrative synthesis and meta-analysis. Public Health. (2016) 136:13–28. 10.1016/j.puhe.2016.02.021 27059370

[23] Mathews C Coetzee N Zwarenstein M Lombard C Guttmacher S Oxman A . A systematic review of strategies for partner notification for sexually transmitted diseases, including HIV/AIDS. Int J STD AIDS. (2002) 13:285–300. 10.1258/0956462021925081 11972932

[24] Taleghani S Joseph-Davey D West SB Klausner HJ Wynn A Klausner JD. Acceptability and efficacy of partner notification for curable sexually transmitted infections in sub-Saharan Africa: a systematic review. Int J STD AIDS. (2019) 30:292–303. 10.1177/0956462418803983 30396318 PMC6441466

[25] Cao W Wong HM Chang C Agudile EP Ekström AM. Behavioral interventions promoting HIV serostatus disclosure to sex partners among HIV-positive men who have sex with men: a systematic review. Int J Public Health. (2019) 64:985–98. 10.1007/s00038-019-01275-4 31250027

[26] Conserve DF Groves AK Maman S. Effectiveness of interventions promoting HIV serostatus disclosure to sexual partners: a systematic review. AIDS Behav. (2015) 19:1763–72. 10.1007/s10461-015-1006-1 25645328 PMC5101233

[27] Phillips AE Gomez GB Boily MC Garnett GP. A systematic review and meta-analysis of quantitative interviewing tools to investigate self-reported HIV and STI associated behaviours in low- and middle-income countries. Int J Epidemiol. (2010) 39:1541–55. 10.1093/ije/dyq114 20630991

[28] Britto C Mehta K Thomas R Shet A. Prevalence and correlates of HIV disclosure among children and adolescents in low- and middle-income countries: a systematic review. J Dev Behav Pediatr. (2016) 37:496–505. 10.1097/DBP.0000000000000303 27262128 PMC5949066

[29] Gabbidon K Chenneville T Peless T Sheared-Evans S. Self-disclosure of HIV status among youth living with HIV: a global systematic review. AIDS Behav. (2020) 24:114–41. 10.1007/s10461-019-02478-9 30924065

[30] Whembolua GL Conserve DF Thomas K Handler L. A systematic review of HIV serostatus disclosure among African immigrants in Europe. J Immigr Minor Health. (2017) 19:947–58. 10.1007/s10903-016-0456-5 27388442

[31] Endalamaw A Assefa Y Geremew D Belete H Dachew BA Belachew A . Disclosure of HIV seropositivity to sexual partner in Ethiopia: a systematic review. Womens Health. (2021) 17:17455065211063021. 10.1177/17455065211063021 34844482 PMC8640980

[32] Mekonnen FA Lakew AM Muchie KF Teshome DF. Sero-positive HIV result disclosure to sexual partner in Ethiopia: a systematic review and meta-analysis. BMC Public Health. (2019) 19:1743. 10.1186/s12889-019-8097-y 31881867 PMC6935207

[33] Yalew M Adane B Kefale B Damtie Y Tadesse SE Molla A. The effect of counseling, antiretroviral therapy and relationship on disclosing HIV positive status to sexual partner among adult HIV patients in Ethiopia: a systematic review and meta-analysis. PLoS ONE. (2021) 16:e0249887. 10.1371/journal.pone.0249887 33886583 PMC8061922

[34] Yehualashet F Tegegne E Tessema M Endeshaw M. Human immunodeficiency virus positive status disclosure to a sexual partner and its determinant factors in Ethiopia: a systematic review and meta-analysis. BMC Infect Dis. (2020) 20:382. 10.1186/s12879-020-05081-9 32471358 PMC7257234

[35] Sullivan KM. Male self-disclosure of HIV-positive serostatus to sex partners: a review of the literature. J Assoc Nurses AIDS Care. (2005) 16:33–47. 10.1016/j.jana.2005.09.005 16536263

[36] Golub SA Indyk D. HIV-infected individuals as partners in prevention: a redefinition of the partner notification process. Soc Work Health Care. (2006) 42:225–35. 10.1300/J010v42n03_14 16687384

[37] Yoshioka MR Schustack A. Disclosure of HIV status: cultural issues of Asian patients. AIDS Patient Care STDS. (2001) 15:77–82. 10.1089/108729101300003672 11224933

[38] UNAIDS. UNAIDS: Cumulative Cross-sectional Cascade for HIV Treatment and Care, China, 2020. Geneva: UNAIDS (2020). Available online at: https://www.aidsdatahub.org/country-profiles/china (accessed June 9, 2022).

[39] Mengjie H Qingfeng C Peng X Ying S. Forging ahead in the “13th Five-Year Plan”, AIDS prevention and control marching towards a new journey—review and prospect of AIDS prevention and control in China. Chin J AIDS STD. (2021) 27:1327–31. [in Chinese]. 10.13419/j.cnki.aids.2021.12.01

[40] World Health Organization. Guidelines on HIV Self-testing and Partner Notification: Supplement to Consolidated Guidelines on HIV Testing Services. Geneva: WHO (2016). 27977094

[41] UNAIDS. AIDSinfo - Global AIDS Response Progress Reporting (GARPR) 2015. Available online at: https://undatacatalog.org/dataset/aidsinfo-global-aids-response-progress-reporting\garpr#:~:text=The%20Global%20AIDS%20Response%20Progress%20Reporting%20data%20consists,reported%20at%20the%20global%20level%20every%20second%20year (accessed July 22, 2022).

[42] Munn Z MS Lisy K Riitano D Tufanaru C. Chapter 5: Systematic reviews of prevalence and incidence. In:Aromataris E Munn Z, editors. JBI Manual for Evidence Synthesis JBI. (2020). Available online at: https://synthesismanualjbiglobal (accessed June 9, 2022).

[43] Page MJ McKenzie JE Bossuyt PM Boutron I Hoffmann TC Mulrow CD . The PRISMA 2020 statement: an updated guideline for reporting systematic reviews. BMJ. (2021) 372:n71. 10.1136/bmj.n71 33782057 PMC8005924

[44] Knoll T Omar MI Maclennan S Hernández V Canfield S Yuan Y . Key steps in conducting systematic reviews for underpinning clinical practice guidelines: methodology of the European Association of Urology. Eur Urol. (2018) 73:290–300. 10.1016/j.eururo.2017.08.016 28917594

[45] Cumpston M Li T Page MJ Chandler J Welch VA Higgins JP . Updated guidance for trusted systematic reviews: a new edition of the Cochrane Handbook for Systematic Reviews of Interventions. Cochrane Database Syst Rev. (2019) 10:Ed000142. 10.1002/14651858.ED000142 31643080 PMC10284251

[46] Civil Code of the People's Republic of China. Beijing: CHINA LEGAL PUBLISHING HOUSE: National People's Congress 2020 May 28 (2020).

[47] Pneumocystis pneumonia–Los Angeles. MMWR Morb Mortal Wkly Rep. (1981) 30:250–2. 6265753

[48] Buscemi N Hartling L Vandermeer B Tjosvold L Klassen TP. Single data extraction generated more errors than double data extraction in systematic reviews. J Clin Epidemiol. (2006) 59:697–703. 10.1016/j.jclinepi.2005.11.010 16765272

[49] JBI. Critical Appraisal Checklist for Analytical Cross Sectional Studies. Jounna Briggs Institute (2017). Available online at: https://jbi.global/critical-appraisal-tools (accessed June 9, 2022).

[50] Zhang C Qian HZ Chen X Bussell S Shen Y Wang H . HIV testing and seroprevalence among couples of people diagnosed with HIV in China: a meta-analysis. PLoS ONE. (2021) 16:e0247754. 10.1371/journal.pone.0247754 33739981 PMC7978381

[51] Higgins JP Thompson SG Deeks JJ Altman DG. Measuring inconsistency in meta-analyses. BMJ. (2003) 327:557–60. 10.1136/bmj.327.7414.557 12958120 PMC192859

[52] Barendregt JJ Doi SA Lee YY Norman RE Vos T. Meta-analysis of prevalence. J Epidemiol Community Health. (2013) 67:974–8. 10.1136/jech-2013-203104 23963506

[53] DerSimonian R Laird N. Meta-analysis in clinical trials. Control Clin Trials. (1986) 7:177–88. 10.1016/0197-2456(86)90046-2 3802833

[54] Egger M Davey Smith G Schneider M Minder C. Bias in meta-analysis detected by a simple, graphical test. BMJ. (1997) 315:629–34. 10.1136/bmj.315.7109.629 9310563 PMC2127453

[55] Rosenfeld RM. Meta-analysis. ORL J Otorhinolaryngol Relat Spec. (2004) 66:186–95. 10.1159/000079876 15467343

[56] Jing W. Heterosexual Behaviors Before and After Diagnosis among Newly Reported HIV/AIDS Cases with Heterosexual Mode of Transmission in Six Districts of Chongqing City (Thesis). Anhui Medical University, Hefei (2020).

[57] Jin-lei Q. An Epidemiological Study of HIV Sexual Transmission in Married Spouses in Yunnan Province (Thesis). Kunming Medical University, Kunming (2012). 22575137

[58] Wanhuai Z Yan C Yueping W Xiang D Yan L. HIV/AIDS spouses inform the status of testing and the influencing factors in Jiangdu District, Yangzhou City. Jiangsu J Prev Med. (2016) 27:436–7. [in Chinese]. 10.13668/j.issn.1006-9070.2016.04.020

[59] Mingjian N. Influence factors of partner notification among people living with HIV/AIDS in Xinjiang. Chin J Public Health. (2011) 27:1508–10. 10.11847/zzggws2013-29-08-03

[60] La-cuo Z Weng-hong L Qiu-shi W Fang L Zhong-hong W Jun H . Analysis of the differences of spousal notification among HIV discordant couples in two key areas of AIDS epidemic in Sichuan Province. Chin J Dis Control Prevent. (2020) 24:1441–6. 10.16462/j.cnki.zhjbkz.2020.12.015

[61] Chechi H Xiang M Wei Z Qinghai H Jing Z Zhenxing C . Resistance factors influencing HIV serostatus disclosure to spouses among married men who have sex with men (MSM) in Shenyang. Chin J AIDS STD. (2018) 24:250−3. 10.13419/j.cnki.aids.2018.03.10

[62] Lifen Z. Sexual risk behavior and associated factors among people living with HIV/AIDS over different time period after being notified as HIV seropositive [thesis]: Peking Union Medical College, Peking (2009).

[63] Mao Y Li X Qiao S Zhao Q Zhou Y Shen Z. Social support, stigma, and HIV disclosure among parents living with HIV in Guangxi, China. AIDS Care. (2018) 30:168–72. 10.1080/09540121.2017.1387639 29020796 PMC6277982

[64] Xiao X Zhao J Tang C Li X Simoni JM Wang H . Psychometric testing of the consequences of an HIV disclosure instrument in Mandarin: a cross-sectional study of persons living with HIV in Hunan, China. Patient Prefer Adherence. (2018) 12:1451–9. 10.2147/PPA.S168571 30147303 PMC6103303

[65] Yan H Cao W Mo P Huan X Wang Z Lin X . Prevalence and associated factors of HIV serostatus disclosure to regular female sex partners among HIV-positive men who have sex with both men and women in China. AIDS Care. (2019) 31:1026–34. 10.1080/09540121.2019.1612002 31046414

[66] Yan X Xu Y Tang W Tucker J Miller W. HIV partner notification among MSM living with HIV in Guangdong Province, China: findings from a cross-sectional study. Sex Transm Infect. (2021) 97:A148–A. 10.1136/sextrans-2021-sti.387

[67] Ding Y Li L Ji G. HIV disclosure in rural China: predictors and relationship to access to care. AIDS Care. (2011) 23:1059–66. 10.1080/09540121.2011.554524 21480006 PMC3157553

[68] Wang L Shan D Chan S Chen H Ge Z Ding G . Disclosure of HIV-positive serostatus to sexual partners and associated factors in southern China. Int J STD AIDS. (2010) 21:685–90. 10.1258/ijsa.2010.010040 21139146

[69] Qiao S Li X Zhou Y Shen Z Tang Z. AIDS impact special issue 2015: interpersonal factors associated with HIV partner disclosure among HIV-infected people in China. AIDS Care. (2016) 28:37–43. 10.1080/09540121.2016.1146397 26899370 PMC4828612

[70] Zang C He X Liu H. Selective disclosure of HIV status in egocentric support networks of people living with HIV/AIDS. AIDS Behav. (2015) 19:72–80. 10.1007/s10461-014-0840-x 24996393 PMC4284146

[71] Huifen Y Yu H Yuhua S Junli H Xiaobo Z Yuecheng Y . Case-control study of the correlation of partner notification and HIV testing with seroconversion of spouses among human immunodeficiency virus sero-discordant couples. Chin J AIDS STD. (2017) 23:898–900+908. 10.13419/j.cnki.aids.2017.10.07

[72] Xiaoyi Y. HIV Transmission between Couples and its Influencing Factors in Guangxi Province (Thesis). Guangxi Medical University, Nanning (2015).

[73] Xu P Liu K Lv F. Analysis on status quo of spousal notification of HIV infectors. Chin J Health Policy. (2011) 4:55–9. 10.3969/j.issn.1647-2982.2011.10.012

[74] Lifen Z. Sexual Risk Behavior and Associated Factors among People Living with HIV/AIDS over Different Time Period after Being Notified as HIV Seropositive (Thesis). Peking Union Medical College, Beijing (2009).

[75] Shaonan l. AIDS Spread in the Families and its Influence Factors (Thesis). University Of Jinan, Jinan (2011).

[76] Min LI. Yun-li LI, Dan Z, Ying-mei Y, Xue-jun C. Analysis of the current situation and influencing factors of the resilience of AIDS patients and infected persons. Pract J Clin Med. (2021) 18:92–5. 10.3969/j.issn.1672-6170.2021.02.026

[77] Ting H Wen-hui C Li-fang D Xiang L Hua J. Change of high-risk behaviours after diagnosis among people infected with HIV by the commercial heterosexual sex, Shaanxi Province. Mod Prevent Med. (2017) 44:2853–6. 10.13419/j.cnki.aids.2017.01.25

[78] Yang R Gui S Xiong Y Gao S Rong Y Yan Y. Characteristics of HIV transmission in MSM fixed with HIV infection in 100 cases. Chin J AIDS STD. (2011) 17:581. [in Chinese]. 10.13419/j.cnki.aids.2011.05.037

[79] Qi-rong Q Feng-lin Z Zheng-dong D Wei Z Ping A Min B . Current situation investigation on HIV/AIDS infection notification and sexual behavior in families with single-positive infection. Occupat Health. (2021) 37:2216–20. 10.13329/j.cnki.zyyjk.2021.0536

[80] Qi W Ding-yong SUN Zhe W Nan MA Ning LI Yan-min MA. Epidemiological analysis of HIV infections *via* sexual transmission between spouses in Henan Province. Chin J Health Educ. (2013) 29:690–2. 10.16168/j.cnki.issn.1002-9982.2013.08.020

[81] Min W. Health Status and Health Service Utilization in a sample of HIV-Positive Men Who Have Sex with Men in Shanghai (thesis). Anhui Medical University, Anhui (2013).

[82] Yinmei Y. HIV Disclosure Patterns and its Associated Factors among People with HIV/AIDS (thesis). Wuhan University, Wuhan (2018).

[83] Shufang H Juan W Jing C Ying S Hong Y Haijing H . HIV status disclosure to sexual partners and its related factors among HIV-positive college students reported from 2016 to 2019 in Beijing. Chin J AIDS STD. (2021) 27:817–21. 10.13419/j.cnki.aids.2021.08.06

[84] Duo S Zeng G Lu W Lifen Z. Self-disclosure of HIV serostatus and influencing factors in rural areas in China. Chin J Dis Control Prevent. (2010) 14:215–8.

[85] Shengyun C Huali W Yuping C Dapeng Z Xiaobin CAO Wenyuan YIN. Study on sextual behavior among HIV patients who are on ART and factors associated with condom use in a prefecture of China. Chin J AIDS STD. (2019) 25:454–8. 10.13419/j.cnki.aids.2019.05.05

[86] Mi G Zeng Y Haoli H Linglin Z Tian L Xiaodong W . A survey on the utilization of AlD/STD related health services in HIV carriers of MSM, Chengdu city. Chin Prevent Med. (2010) 11:328–31. 10.16506/j.109-639.2010.04.020.328

[87] Yi Y KongIai Z Zerong L Keron W. Self-disclosure of HIV status to significant others. Chin J Dis Control Prevent. (2005) 9:202–4.

[88] Naipeng L. The Risk of HIV Transmission via Sex and its Correlates among HIV-positive Men who Have Sex with Men (thesis). Anhui Medical University, Anhui (2017).

[89] Guanghua L Nengxiu L Yuejiao Z Zhiyong S Qiongguang H Jiaxiao J . Influencing factors of HIV positive serostatus disclosure to the regular female sexual partners by HIV infected men who have sex with men in Guangxi. Chin J AIDS STD. (2020) 26:949–53. 10.13419/j.cnki.aids.2020.09.10

[90] Liangjia Z. Study on HIV Infection and Associated Factors in MSM and HIV Infected Individuals' Spouses (thesis). Southeast University, Nanjing (2014).

[91] Wei J. Willingness and its Correlations of Promoting HIV Testing of Sexual Partners by Self-testing Kits among HIV-positive MSM in Guangzhou (thesis). Guangdong Pharmaceutical University, Guangdong (2017).

[92] Dexiang C. Investigation and study of the current situation and influencing factors of those with positive HIV test results in the grass-roots community. Jiankang Bidu. (2010) 29. [in Chinese].

[93] Yaya I Saka B Landoh DE Patchali PM Patassi AA Aboubakari AS . HIV status disclosure to sexual partners, among people living with HIV and AIDS on antiretroviral therapy at Sokodé regional hospital, Togo. PLoS ONE. (2015) 10:e0118157. 10.1371/journal.pone.0118157 25658105 PMC4320091

[94] King R Katuntu D Lifshay J Packel L Batamwita R Nakayiwa S . Processes and outcomes of HIV serostatus disclosure to sexual partners among people living with HIV in Uganda. AIDS Behav. (2008) 12:232–43. 10.1007/s10461-007-9307-7 17828450

[95] Przybyla SM Golin CE Widman L Grodensky CA Earp JA Suchindran C. Serostatus disclosure to sexual partners among people living with HIV: examining the roles of partner characteristics and stigma. AIDS Care. (2013) 25:566–72. 10.1080/09540121.2012.722601 23020136 PMC3622199

[96] Trinh TT Yatich N Ngomoa R McGrath CJ Richardson BA Sakr SR . Partner disclosure and early CD4 response among HIV-infected adults initiating antiretroviral treatment in Nairobi Kenya. PLoS ONE. (2016) 11:e0163594. 10.1371/journal.pone.0163594 27711164 PMC5053490

[97] Simbayi LC Zungu N Evans M Mehlomakulu V Kupamupindi T Mafoko G . HIV Serostatus disclosure to sexual partners among sexually active people living with HIV in South Africa: results from the 2012 National Population-Based Household Survey. AIDS Behav. (2017) 21:82–92. 10.1007/s10461-015-1278-5 26767538

[98] Galletly CL Glasman LR Pinkerton SD Difranceisco W. New Jersey's HIV exposure law and the HIV-related attitudes, beliefs, and sexual and seropositive status disclosure behaviors of persons living with HIV. Am J Public Health. (2012) 102:2135–40. 10.2105/AJPH.2012.300664 22994175 PMC3477954

[99] Partner Notification Services. Peking (2019).

[100] Chen G. Discussion on the duty of AIDS patient to inform and the partner's right to know. Legal Syst Soc. (2021) 10:20–1, +34.[in Chinese].

[101] UNAIDS Reference Group on HIV Human Rights. Statement on Criminalization of HIV Transmission and Exposure. Geneva: UNAIDS Reference Group on HIV and Human Rights (2008). Available online at: http://data.unaids.org/pub/Report/2009/20090303_hrrefgroupcrimexposure_en.pdf (accessed June 30, 2022).

[102] AIDS prevention and control regulations (2019). Chin J AIDS STD. (2019) 25:435–8. [in Chinese].

[103] He H Xu P Xin Q Zeng J Zhang L Sun D . Study on spousal notification in HIV discordant couples and associated factors in four provinces of China. Zhonghua Liu Xing Bing Xue Za Zhi. (2015) 36:565–8. 10.3760/cma.j.issn.0254-6450.2015.06.006 26564625

[104] Xu P ZG Liu KM. Policy Analysis on Complusory Sopusal Notification among HIV Discordant Couples. National Seminar on Health Law; Shanghai (2011), p. 181–6. [in Chinese].

[105] Obermeyer CM Baijal P Pegurri E. Facilitating HIV disclosure across diverse settings: a review. Am J Public Health. (2011) 101:1011–23. 10.2105/AJPH.2010.300102 21493947 PMC3093267

[106] Dongmei Y Xiujun Z. Stigma status of HIV/AIDS patients. Chin J AIDS STD. (2016) 22:740−2. 10.13419/j.cnki.aids.2016.09.2019708868

[107] Fife BL Wright ER. The dimensionality of stigma: a comparison of its impact on the self of persons with HIV/AIDS and cancer. J Health Soc Behav. (2000) 41:50–67. 10.2307/2676360 10750322

[108] Hsieh MT Chen JS Lin CY Yen CF Griffiths MD Huang YT. Measurement invariance of the sexual orientation microaggression inventory across LGB males and females in Taiwan: bifactor structure fits the best. Int J Environ Res Public Health. (2021) 18:2–12. 10.3390/ijerph182010668 34682410 PMC8536138

[109] Johnson P. Homosexuality and the European Court of Human Rights. London: Routledge (2012). 10.4324/9780203096581

[110] Finck M. The role of human dignity in gay rights adjudication and mediation: a comparative perspective. Int J Const Law. (2016) 14:26–53. 10.1093/icon/mow009

[111] Chi Y Huang D Pachankis J Valimaki M Shen Y Li X. Internalized Sexual minority stigma is associated with HIV testing behavior among chinese men who have sex with men: a cross-sectional study. J Assoc Nurses AIDS Care. (2021) 32:578–88. 10.1097/JNC.0000000000000205 35137720

[112] Wu W Yan X Zhang X Goldsamt L Chi Y Huang D . Potential HIV transmission risk among spouses: marriage intention and expected extramarital male-to-male sex among single men who have sex with men in Hunan, China. Sex Transm Infect. (2020) 96:151–6. 10.1136/sextrans-2018-053906 31171593 PMC7035676

[113] Zola EK Gifudu GM Henry E Bernier A Masangu HM Abadie A . Factors associated with HIV voluntary disclosure of people living with HIV to their steady sexual partner in the Democratic Republic of the Congo: results from a community-based participatory research. Pan Afr Med J. (2014) 19:276. 10.11604/pamj.2014.19.276.5304 25870731 PMC4391903

[114] Loukid M Abadie A Henry E Hilali MK Fugon L Rafif N . Factors associated with HIV status disclosure to one's steady sexual partner in PLHIV in Morocco. J Community Health. (2014) 39:50–9. 10.1007/s10900-013-9739-0 23913104

[115] Hu L-F Zhang Y-J Li M-Q Feng X-X Liu J Ni J . Serostatus disclosure and associated factors among people living with HIV/AIDS (PLWHA) in Liuzhou City, China. Chin J Dis Control Prevent. (2014) 18:185–9.

[116] Mengjun T Jaling H. Study on the status quo and influence factors of aids transmission between spouses in China. China Popul Today. (2009) 16:39–44, 19–28.

[117] Ting-ting C Zi-han G Ya-jing Z Yi-peng S Wei-ping C Yan G. HIV disclosure to family members and its influencing factors among people with HIV/AIDS. Chin J Dis Control Prevent. (2015) 19:1255–9. 10.16462/j.cnki.zhjbkz.2015.12.018

[118] Wu J Wu GH Zhang W Wu ZY. Characteristics of newly reported HIV/AIDS cases with heterosexual mode of transmission in six districts of Chongqing city. Zhonghua Liu Xing Bing Xue Za Zhi. (2020) 41:919–23. 10.3760/cma.j.cn112338-20191211-00873 32564560

[119] Campbell A. The morning after the night before: affective reactions to one-night stands among mated and unmated women and men. Hum Nat. (2008) 19:157–73. 10.1007/s12110-008-9036-2 26181462

[120] Zhang K Zhao J Li X Chen X Wang H Williams AB . Perceived facilitators and barriers regarding partner notification in people living with HIV in Hunan, China: a qualitative study from the patient perspective. J Assoc Nurses AIDS Care. (2019) 30:658–67. 10.1097/JNC.0000000000000093 31574528

[121] Deribe K Woldemichael K Wondafrash M Haile A Amberbir A. Disclosure experience and associated factors among HIV positive men and women clinical service users in Southwest Ethiopia. BMC Public Health. (2008) 8:81. 10.1186/1471-2458-8-81 18312653 PMC2275263

[122] Kiene SM Dove M Wanyenze RK. Depressive symptoms, disclosure, HIV-related Stigma, and coping following HIV testing among outpatients in Uganda: a daily process analysis. AIDS Behav. (2018) 22:1639–51. 10.1007/s10461-017-1953-9 29081046 PMC5903948

[123] Amoran OE. Predictors of disclosure of sero-status to sexual partners among people living with HIV/AIDS in Ogun State, Nigeria. Niger J Clin Pract. (2012) 15:385–90. 10.4103/1119-3077.104507 23238184

[124] Antelman G Smith Fawzi MC Kaaya S Mbwambo J Msamanga GI Hunter DJ . Predictors of HIV-1 serostatus disclosure: a prospective study among HIV-infected pregnant women in Dar es Salaam, Tanzania. AIDS. (2001) 15:1865–74. 10.1097/00002030-200109280-00017 11579250 PMC6261328

[125] Wu AH TZ Wang W. Query answer over inconsistent database with credible annotation. J Softw. (2012) 23:1167–82. 10.3724/SP.J.1001.2012.04079

[126] Wu P Dong WM Rou K Dong W Zhou C Chen X . HIV-positive clients of female sex workers in Hunan Province, China: a mixed methods study assessing sexual relationships and risk behavior by type of partner. BMC Public Health. (2019) 19:1129. 10.1186/s12889-019-7446-1 31420032 PMC6698027

[127] Marks G Burris S Peterman TA. Reducing sexual transmission of HIV from those who know they are infected: the need for personal and collective responsibility. AIDS. (1999) 13:297–306. 10.1097/00002030-199902250-00001 10199219

[128] UNAIDS: Understanding fast-track: accelerating action to end the AIDS epidemic by 2030. Available online at: https://www.unaids.org/sites/default/files~media_asset201506_JC2743_Understanding_FastTrack_en.pdf (accessed June 29, 2022).

