# Supplementary material for: The proportion of HIV disclosure to sexual partners among people diagnosed with HIV in China: A systematic review and meta-analysis
Source: Front Public Health. 2022 Oct 17;10:1004869. doi: 10.3389/fpubh.2022.1004869 (PMC9620859; doi:10.3389/fpubh.2022.1004869)
Supplement: Supplementary Table S1 — The search terms used in the databases and the number of results. [file Data_Sheet_1.zip › S1 Table..docx]

**S1** **Table. The search terms used in the databases and the** **number of results.**

| **Databases** | **Search terms** | **Number of results** |
| --- | --- | --- |
| Four English databases (search in English) | | |
| PubMed | #1:(((HIV[MeSH Terms]) OR (HIV infections[MeSH Terms])) OR (Acquired Immunodeficiency Syndrome[MeSH Terms])) OR (AIDS)  #2:(((((((Self Disclosure[MeSH Terms]) OR (Disclosure[MeSH Terms])) OR (HIV Disclosure)) OR (HIV serostatus disclosure)) OR (partner disclosure)) OR (partner notification)) OR (disclos*)) OR (expos*)  #3:(China) OR (chinese)  #1 AND #2 AND #3 | 1225 |
| Web of Science | ((TS=(HIV OR Human immunodeficiency virus OR HIV infections OR AIDS OR Acquired Immunodeficiency Syndrome)) AND TS=(Self Disclosure OR Disclosure OR HIV Disclosure OR HIV serostatus disclosure OR partner disclosure OR partner notification OR disclos* OR expos*)) AND TS=(China OR Chinese) | 1537 |
| Embase | ('human immunodeficiency virus'/exp OR 'human immunodeficiency virus' OR 'human immunodeficiency virus'/exp OR 'hiv infections'/exp OR 'hiv infections' OR (('hiv'/exp OR hiv) AND ('infections'/exp OR infections)) OR 'acquired immune deficiency syndrome'/exp OR 'acquired immune deficiency syndrome')  AND  ('hiv serostatus disclosure' OR (('hiv'/exp OR hiv) AND serostatus AND ('disclosure'/exp OR disclosure)) OR 'partner notification'/exp OR 'partner notification' OR 'self disclosure'/exp OR 'self disclosure')  AND  ('china'/exp OR china OR 'chinese'/exp OR chinese) | 60 |
| Cochrane  Library | ((HIV OR Human immunodeficiency virus OR HIV infections OR AIDS OR Acquired Immunodeficiency Syndrome (AIDS) )):ti,ab,kw AND ((Truth Disclosure OR Self Disclosure OR Disclosure OR HIV Disclosure OR HIV serostatus disclosure OR partner disclosure OR disclos* OR expos* OR reveal* OR partner notification)):ti,ab,kw AND ((China OR Chinese)):ti,ab,kw | 84 |
| Four Chinese databases (search in Chinese) | | |
| Wan Fang | Theme: (HIV OR HIV infector OR HIV infection OR HIV serum positive OR HIV positive OR AIDS OR AIDS patient OR AIDS OR Acquired immunodeficiency syndrome OR HIV/AIDS patient) and Theme:(sexual partner notification OR spouse notification OR (sexual partner AND notification) OR (spouse AND notification)) | 212 |
| the China National Knowledge Internet | (Theme：HIV+HIV infector+HIV infection+HIV serum positive+HIV positive+HIV patient+AIDS+AIDS patient+AIDS infector+AIDS+Acquired immunodeficiency syndrome+HIV/AIDS patients) AND ((Theme：sexual partner notification+spouse notification) OR (Theme：sexual partner*notification) OR (Theme：spouse*notification)) | 488 |
| Sino Med | #1:(("HIV infection"[All fields] OR "HTLV-Ⅲ infection"[All fields] OR "HTLV-Ⅲ-LAV infection"[All fields] OR "Human type Ⅲ infection with T-lymphotropic virus"[All fields] OR "HIV infection"[MeSH Terms]) OR ("AIDS"[All fields] OR "Acquired immunodeficiency syndrome"[All fields] OR "Acquired immunodeficiency syndrome"[All fields] OR "Acquired immunodeficiency syndrome"[MeSH Terms])  OR ("HIV serum positive"[All fields] OR "AIDS serum conversion"[All fields] OR "AIDS serum positive"[All fields] OR "anti-HIV positive"[All fields] OR "HIV antibody positive"[All fields] OR "HIV serum conversion"[All fields] OR "HTLV-Ⅲserum conversion"[All fields] OR "HTLV-ⅢAIDS serum positive"[All fields] OR "HIV serum positive"[MeSH Terms]) OR "HIV positive"[All fields])  #2:("sexual partner notification"[All fields] OR "spouse notification"[All fields] OR"notification"[All fields]) OR "notification"[All fields])  #3:("spouse"[All fields] OR "domestic partner"[All fields] OR "husband"[All fields] OR "Married people"[All fields] OR "wife"[All fields] OR "spouse"[MeSH Terms]) OR "sexual partner"[All fields]  #1 AND (#2 AND #3) | 54 |
| VIP data | （U=(HIV OR HIV infector OR HIV infection OR HIV serum positive OR HIV positive OR AIDS OR AIDS patients OR AIDS OR Acquired immunodeficiency syndrome OR HIV/AIDS patients)) AND (U=(sexual partner notification OR spouse notification OR (sexual partner AND notification) OR (spouse AND notification))) | 38 |
