# Supplementary material for: The proportion of HIV disclosure to sexual partners among people diagnosed with HIV in China: A systematic review and meta-analysis
Source: Front Public Health. 2022 Oct 17;10:1004869. doi: 10.3389/fpubh.2022.1004869 (PMC9620859; doi:10.3389/fpubh.2022.1004869)
Supplement: Supplementary Table S1 — The search terms used in the databases and the number of results. [file Data_Sheet_1.zip › S3 Table. Subgroup analyses..docx]

**S3 Table. Subgroup analyses of HIV disclosure to sexual partners.**

| **Subgroups** | **No. of studies** | **ES (95% CI)** | **I^2^, %** | **P value for heterogeneity** |
| --- | --- | --- | --- | --- |
| **Subgroup1: HIV disclosure to different types of sexual partners** | | | | |
| unclassified | 14 | 0.63 (0.45,0.81) | 99.7 | 0.000 |
| regular sexual partner | 31 | 0.66 (0.59,0.73) | 99.1 | 0.000 |
| casual sexual partner | 9 | 0.20 (0.08,0.33) | 98.0 | 0.000 |
| **Subgroup2: HIV disclosure at different diagnosis times** | | | | |
| on the day of diagnosis | 3 | 0.57 (0.45,0.69) | 89.5 | 0.000 |
| within a month of diagnosis | 4 | 0.62 (0.42,0.82) | 97.7 | 0.000 |
| at a month after diagnosis | 4 | 0.39 (0.02,0.77) | 99.5 | 0.000 |
| **Subgroup3: HIV disclosure to different types of sexual partners among the MSM** | | | | |
| regular male sexual partner | 6 | 0.47 (0.29,0.65) | 97.8 | 0.000 |
| regular female sexual partner | 3 | 0.49 (0.33,0.65) | 96.0 | 0.000 |
| spouse | 7 | 0.48 (0.18,0.78) | 99.2 | 0.000 |
| casual sexual partner | 4 | 0.34 (0.19,0.49) | 95.7 | 0.000 |
