# Supplementary figures and images for: The proportion of HIV disclosure to sexual partners among people diagnosed with HIV in China: A systematic review and meta-analysis
Source: Front Public Health. 2022 Oct 17;10:1004869. doi: 10.3389/fpubh.2022.1004869 (PMC9620859; doi:10.3389/fpubh.2022.1004869)

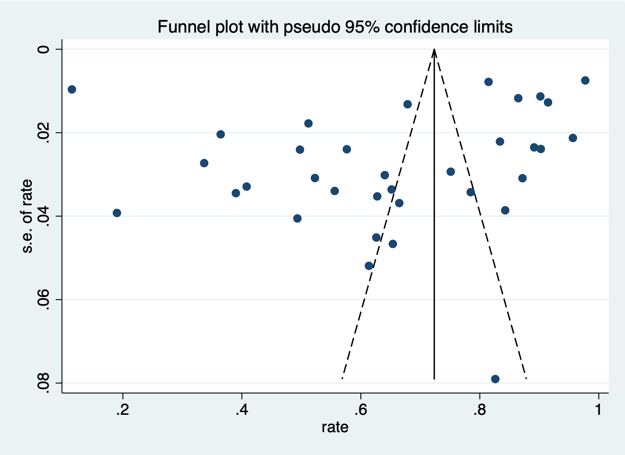

Supplement: Supplementary Table S1 — The search terms used in the databases and the number of results. [file Data_Sheet_1.zip › S4 Fig. Funnel plot of HIV disclosure to sexual partners among PDWH.jpg]

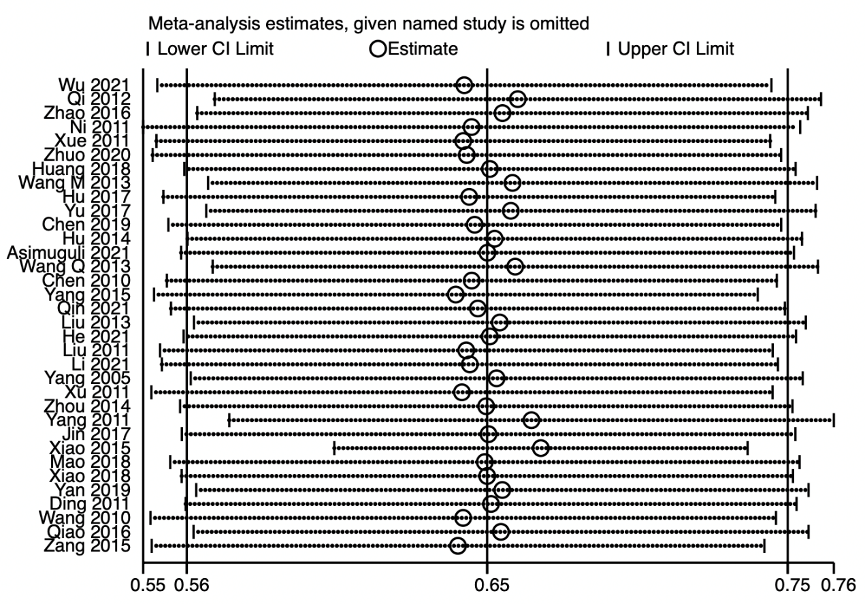

Supplement: Supplementary Table S1 — The search terms used in the databases and the number of results. [file Data_Sheet_1.zip › S5 Fig. Sensitivity analysis for the pooled proportion of HIV disclosure to sexual partners.jpg]
