# Supplementary material for: The proportion of HIV disclosure to sexual partners among people diagnosed with HIV in China: A systematic review and meta-analysis
Source: Front Public Health. 2022 Oct 17;10:1004869. doi: 10.3389/fpubh.2022.1004869 (PMC9620859; doi:10.3389/fpubh.2022.1004869)
Supplement: Supplementary Table S1 — The search terms used in the databases and the number of results. [file Data_Sheet_1.zip › S2 Table.doc]

**S2 Table. Assessment of methodological quality of analytical cross-sectional studies(n=44).**

| **Authors year** | **Q1** | **Q2** | **Q3** | **Q4** | **Q5** | **Q6** | **Q7** | **Q8** | **Score** |
| --- | --- | --- | --- | --- | --- | --- | --- | --- | --- |
| Wu 2021 | Y | Y | Y | Y | NA | NA | N | Y | 5 |
| Qi 2012 | Y | Y | Y | Y | Y | Y | Y | Y | 8 |
| Zhao 2016 | Y | Y | Y | Y | NA | Y | Y | Y | 7 |
| Ni 2011 | Y | Y | Y | Y | Y | N | Y | Y | 7 |
| Xue 2011 | Y | Y | Y | Y | N | N | Y | Y | 6 |
| Zhuo 2020 | Y | Y | Y | Y | Y | Y | Y | Y | 8 |
| Huang 2018 | Y | Y | Y | Y | NA | NA | Y | Y | 6 |
| Wang M 2013 | Y | Y | Y | Y | Y | Y | Y | Y | 8 |
| Hu 2017 | Y | Y | Y | Y | Y | Y | Y | Y | 8 |
| Yu 2017 | Y | Y | Y | Y | Y | Y | Y | Y | 8 |
| Shan 2010 | Y | Y | Y | Y | Y | Y | Y | Y | 8 |
| Chen 2019 | Y | Y | Y | Y | Y | Y | Y | Y | 8 |
| Hu 2014 | Y | Y | Y | Y | Y | Y | Y | Y | 8 |
| Gao 2010 | Y | Y | Y | Y | Y | Y | Y | Y | 8 |
| Asimuguli 2021 | Y | Y | Y | Y | Y | Y | Y | Y | 8 |
| Wang Q 2013 | Y | Y | Y | Y | NA | NA | N | Y | 5 |
| Lan 2020 | Y | Y | Y | Y | Y | Y | Y | Y | 8 |
| Chen 2010 | Y | N | Y | Y | N | N | Y | Y | 5 |
| Yang 2015 | Y | Y | Y | Y | Y | Y | Y | Y | 8 |
| Qin 2021 | Y | Y | Y | Y | NA | NA | Y | Y | 6 |
| Mi 2010 | Y | Y | Y | Y | NA | NA | Y | Y | 6 |
| Liu 2013 | Y | Y | Y | Y | Y | Y | Y | Y | 8 |
| He 2021 | Y | Y | Y | Y | Y | Y | Y | Y | 8 |
| Liu 2011 | Y | Y | Y | Y | Y | N | Y | Y | 7 |
| Li 2021 | Y | Y | Y | Y | Y | Y | Y | Y | 8 |
| Yang 2005 | Y | Y | Y | Y | NA | NA | Y | Y | 6 |
| Xu 2011 | Y | Y | Y | Y | NA | NA | Y | Y | 6 |
| Zhou 2014 | Y | Y | Y | Y | Y | Y | Y | Y | 8 |
| Yang 2011 | Y | Y | Y | Y | NA | NA | Y | U | 5 |
| Yang 2018 | Y | Y | Y | Y | Y | Y | Y | Y | 8 |
| Jin 2017 | Y | Y | Y | Y | Y | Y | Y | Y | 8 |
| Liu 2017 | Y | Y | Y | Y | Y | Y | Y | Y | 8 |
| Xiao 2015 | Y | Y | Y | Y | NA | NA | Y | Y | 6 |
| Mao 2018 | Y | Y | Y | Y | Y | Y | N | Y | 7 |
| Xiao 2018 | Y | Y | Y | Y | Y | Y | Y | Y | 8 |
| Yan 2019 | Y | Y | Y | Y | Y | Y | Y | Y | 8 |
| Yan 2021 | Y | Y | Y | Y | NA | NA | Y | Y | 6 |
| Ding 2011 | Y | Y | Y | Y | Y | Y | Y | Y | 8 |
| Wang 2010 | Y | Y | Y | Y | Y | N | Y | Y | 7 |
| Qiao 2016 | Y | Y | Y | Y | Y | NA | Y | Y | 7 |
| Zang 2015 | Y | Y | Y | Y | NA | NA | Y | Y | 6 |
| Chen 2014 | Y | Y | Y | Y | Y | Y | Y | Y | 8 |
| Chen 2013 | Y | Y | Y | Y | Y | Y | Y | Y | 8 |
| Zhang 2009 | Y | Y | Y | Y | Y | Y | Y | Y | 8 |

Q= Question; Y=Yes; N= No; NA=Not Applicable; U=Unclear

Q1: Were the criteria for inclusion in the sample clearly defined?

Q2: Were the study subjects and the setting described in detail?

Q3: Was the exposure measured in a valid and reliable way?

Q4: Were objective, standard criteria used for measurement of the condition?

Q5: Were confounding factors identified?

Q6: Were strategies to deal with confounding factors stated?

Q7: Were the outcomes measured in a valid and reliable way?

Q8: Was appropriate statistical analysis used?
